# g-C_3_N_4_ modified flower-like CuCo_2_O_4_ array on nickel foam without binder for high-performance supercapacitors

**DOI:** 10.1039/d4ra07645a

**Published:** 2025-01-03

**Authors:** Lina Ma, Xiaojie He, Shasha He, Shirui Yu, Song Zhang, Yongming Fu

**Affiliations:** a Department of Food Science and Engineering, Moutai Institute Zunyi 564507 China; b School of Physics and Electronic Engineering, State Key Laboratory of Quantum Optics and Quantum Optics Devices, Institute of Laser Spectroscopy, Shanxi University Taiyuan 030006 China fuyongming@sxu.edu.cn

## Abstract

This study investigates the impact of integrating g-C_3_N_4_ into CuCo_2_O_4_ electrodes on electrochemical performance working as binder-free electrodes. Flower-like CuCo_2_O_4_ nanostructures on nickel foam are decorated with few-layer g-C_3_N_4_ using a secondary hydrothermal process. The hierarchical g-C_3_N_4_/CuCo_2_O_4_ nanoflower electrode demonstrates a specific capacity of 247.5 mA h g^−1^ at a current density of 1 A g^−1^, while maintaining a capacity of 87.0 mA h g^−1^ at a heightened current density of 5 A g^−1^. Notably, this electrode exhibited remarkable durability, retaining 98% of its capacity after 1000 cycles. The g-C_3_N_4_/CuCo_2_O_4_ heterostructure shows promise for high-performance energy storage devices.

## Abstract 2

Flower-like g-C_3_N_4_/CuCo_2_O_4_ structures have been successfully established by decorating g-C_3_N_4_ on CuCo_2_O_4_ nanostructures, demonstrating excellent specific capacity and cyclic stability as a pseudocapacitance electrode.

## Graphical abstract

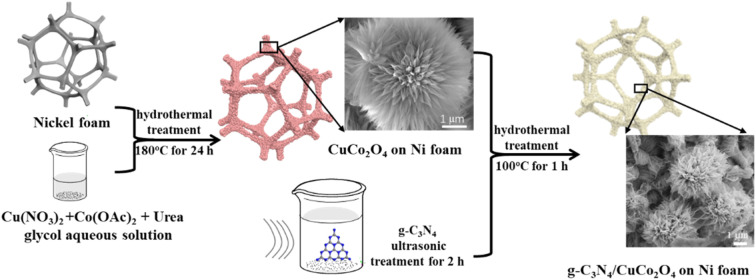

## 1. Introduction

The problems related to environmental pollution from fossil fuel energy sources, along with the swift rise in demand for portable electronic devices, have posed considerable challenges for energy storage materials. Consequently, researchers have been motivated to explore energy storage materials that provide higher power density, greater charge–discharge speed, and outstanding longevity.^1,2^ Electrochemical energy storage devices generally include rechargeable batteries and supercapacitors. In contrast to traditional batteries, which depend on chemical reactions for energy storage and release, supercapacitors operate based on a physical mechanism known as electric double-layer capacitance. One of the main advantages of supercapacitors lies in their affordability. The components and production techniques utilized in creating these devices are relatively simple, leading to lower manufacturing costs compared to batteries.^3^

Supercapacitors are appealing due to their cost-effectiveness, simplicity of manufacturing, impressive power density, high charge–discharge speed, and outstanding longevity. These features make them extremely well-suited for meeting the demands of modern portable gadgets, such as wearable technology.^4^ Energy storage in electric double-layer capacitors is limited to the interface of the electrode material, resulting in a constrained capacity. For instance, the use of activated carbon as electrodes results in a decrease in the discharging capacity of the carbon electrodes (70–250 F g^−1^).^5^ Nevertheless, faradaic capacitor materials offer advantages over electrochemical double-layer capacitors by not only storing electric double-layer energy but also maintaining charge through electron transfer at active electrode electrochemical sites during fast redox reactions.^6,7^ While serving as effective faradaic capacitor electrodes, bimetallic oxides with multiple oxidation states exhibit superior reversible capacity, structural durability, and electrical stability compared to single metal oxides. CuCo_2_O_4_, a bi-transition metal oxide that combines the advantageous properties of both copper and cobalt oxides, displays good electrical conductivity and excellent electrochemical activity. Despite these benefits, the practical application of CuCo_2_O_4_ in supercapacitors is still hindered by its limited surface area and inherent poor cycling stability. To date, many efforts have been devoted to the design of spinel CuCo_2_O_4_ electrodes with diverse morphologies such as nanobelts,^8^ nanograss,^9^ nanowires,^10,11^ and nanosheets.^12,13^ Moreover, several CuCo_2_O_4_-based nanocomposites have been explored through a variety of synthetic routes, such as CuCo_2_O_4_/CuO,^14^ CuCo_2_O_4_/MnCo_2_O_4_ heterostructures,^15^ CuCo_2_O_4_@CQDs,^16^ CuCo_2_S_4_/CuCo_2_O_4_,^17,18^ CuCo_2_O_4_@Co_3_O_4_,^19^ CuCo_2_O_4_@CuCo_2_S_4_@Co(OH)_2_,^20^ and CuCo_2_O_4_/rGO.^21^

g-C_3_N_4_ possesses a two-dimensional sheet structure and demonstrates exceptional performance in semiconductor applications, photocatalysis, environmental science, and other fields. This work presents a novel approach aimed at improving the electrochemical performance of CuCo_2_O_4_ nanoflowers on nickel foam through the coating of g-C_3_N_4_ nanosheets, generating synergistic effects between the bimetal oxide and the metal-free g-C_3_N_4_. The layered structure of g-C_3_N_4_ facilitates the surface area of the electrode, and the good chemical stability of g-C_3_N_4_ helps in improving cycling capacity retention. An asymmetrical supercapacitor is fabricated to demonstrate the application potential of the g-C_3_N_4_/CuCo_2_O_4_ nanocomposite. This work not only highlights the potential of g-C_3_N_4_ as an effective coating material for transition metal oxides but also provides valuable insights into the design of high-performance electrode materials for next-generation supercapacitors.

## 2. Materials and methods

### 2.1 Synthesis of nanoflower CuCo_2_O_4_ on nickel foam carrier

**Fig. 1:**
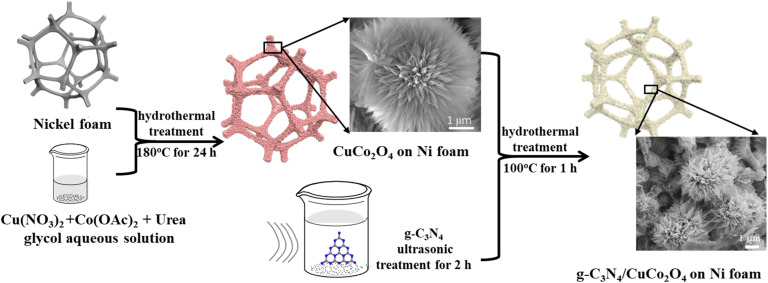
Schematic diagram of the synthetic procedure.

All chemicals were of reagent quality and used without any additional refinement. The nickel foam was cut to a size of 1 cm × 1 cm and then alternately washed with ultrasonic assistance with acetone, 3 M HCl, deionized water, and ethanol for 10 minutes each. Finally, the nickel foam was dried in vacuum at 50 °C for 10 hours, weighed, and stored. The synthetic route described in the following is shown in Fig. 1. The precursor solution for synthesizing CuCo_2_O_4_ was prepared by dissolving copper nitrate trihydrate (0.2416 mg), cobalt acetate tetrahydrate (0.4982 mg), and urea (0.3600 mg) in a solvent under continuous stirring until a uniform blue solution with a pH value of 5 was obtained. To control the morphology of CuCo_2_O_4_ nanostructure by adjusting the solubility and wetting effect, 10 mL of ethylene glycol and 20 mL of deionized water were mixed as a solvent. The cleaned nickel foam was then immersed in the precursor solution and transferred in a 50 mL PTFE-lined stainless-steel autoclave. The autoclave was sealed and heated at 120 °C in a blast drying oven for 12 hours to complete the reaction. After cooling down to room temperature, the CuCo_2_O_4_ electrode material on the nickel foam was obtained by rinsing with deionized water and drying at 60 °C in vacuum.

### 2.2 Synthesis of nanoflower g-C_3_N_4_/CuCo_2_O_4_ on nickel foam

Nanoflower g-C_3_N_4_/CuCo_2_O_4_ was synthesized by coating few-layer g-C_3_N_4_ on the surface of CuCo_2_O_4_. The g-C_3_N_4_ powder was obtained by annealing urea in air at 550 °C for 2 hours. Then, 10 mg of g-C_3_N_4_ powder was added in 30 mL of deionized water in a 50 mL volumetric flask and dispersed ultrasonically for 1 hour. After sitting undisturbed for one day, a suspension with a g-C_3_N_4_ concentration of 0.116 g L^−1^ was obtained in the supernatant, indicating a production rate of ∼30%. Then, the as-prepared CuCo_2_O_4_-coated nickel foam was immersed in 20 mL of the g-C_3_N_4_ suspension, sealed in an air oven, and heated at 120 °C for 12 hours. To study the effect of g-C_3_N_4_ concentration on the electrochemical performance, each of 2, 5, 10, and 15 mL of g-C_3_N_4_ suspension was diluted with deionized water to 20 mL to repeat the above experiment. Finally, the nickel foam was rinsed with deionized water and dried at 60 °C to obtain nanoflower g-C_3_N_4_/CuCo_2_O_4_ on nickel foam.

### 2.3 Characterization

The morphologies and compositions of the samples were analyzed using a field-emission scanning electron microscope (FE-SEM, SUPRA 40, Zeiss, Germany) and a transmission electron microscope (TEM, Tecnai G20, FEI, USA). Surface compositions were examined through X-ray photoelectron spectroscopy (XPS, Escalab 250Xi, Thermo Fisher Scientific, USA). To avoid charging effect, all spectra were referenced to the C–C bond at 284.8 eV in the C 1s spectrum. The samples' crystallographic structure was analyzed using X-ray diffraction (XRD, D8-Discovery, Brucker, 40 kV, 15 mA, Cu Kα, *λ* = 1.5406 Å), while the functional groups in the samples were investigated through Fourier transform infrared (FTIR) spectroscopy (Nicolet-5700, Thermo Fisher, USA).

The electrochemical characteristics of the samples were examined using an electrochemical workstation (CHI 760E, CH Instruments, China) using a standard three-electrode electrochemical setup. g-C_3_N_4_/CuCo_2_O_4_ on nickel foam was utilized directly as the working electrode without using conductive agents or binders. A Hg/HgO electrode and a platinum plate served as the reference electrode and the counter electrode, respectively. All the electrochemical tests were conducted in a 6.0 mol L^−1^ KOH aqueous solution at ambient temperature. Various scan rates were employed for cyclic voltammetry (CV) tests, while galvanostatic charge–discharge (GCD) tests were conducted at different current densities. The specific capacitances (*C*) were determined from the GCD discharge curves by formula (1):

1 *C* = *I* × Δ*t*/(Δ*V* × *m*)

where *I* represents the discharge current in amperes (A), *t* denotes the duration of discharge in seconds (s), Δ*V* indicates the voltage range in volts (V), and *m* refers to the mass of the active materials measured in grams (g).

The calculation of the energy density and power density for the electrodes was performed using formulas (2) and (3):2
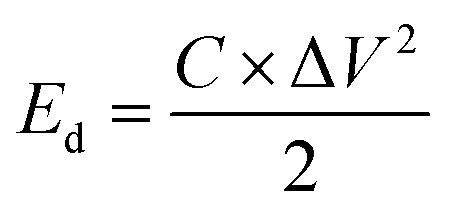
3
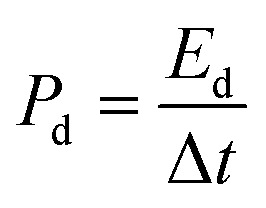


In the equation for energy storage systems, the energy density, denoted as *E*_d_ and measured in watt-hours per kilogram (W h kg^−1^), plays a crucial role in determining the amount of energy that can be stored in a given mass of material. Specific capacitance, represented as *C* and expressed in farads per gram (F g^−1^), is another significant parameter that reflects the ability of a material to store electrical charge. Additionally, the potential range, indicated as Δ*V* and measured in volts (V), outlines the voltage limits within which the energy storage device operates effectively. Furthermore, power density, denoted as *P*_d_ and expressed in watts per kilogram (W kg^−1^), quantifies the rate at which energy can be delivered per unit mass. Lastly, the discharge time, denoted as Δ*t* and measured in seconds (s), indicates the duration required for the device to release its stored energy.

Electrochemical impedance spectroscopy (EIS) was conducted under a measured open voltage as the initial voltage and an amplitude of 5 mV with frequency scanning from 0.01 Hz to 100 kHz.

## 3. Results and discussion

**Fig. 2:**
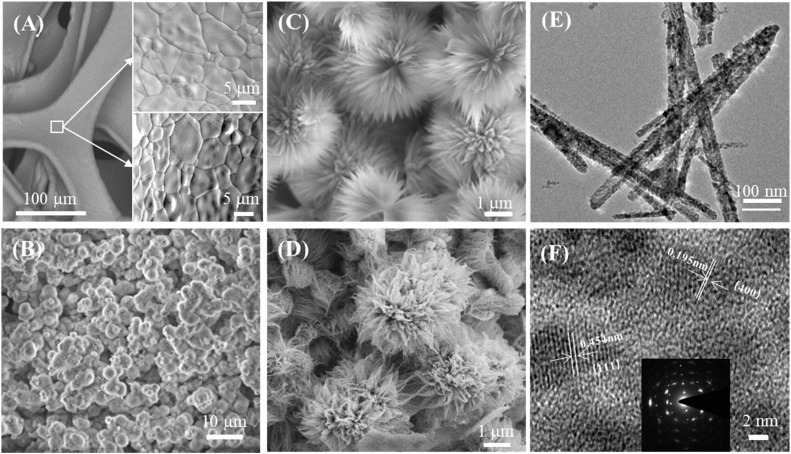
(A) SEM images of the nickel foam surface before and after acid treatment. (B) The CuCo_2_O_4_ nanoflower structure deposited on the nickel foam. (C) Magnified SEM image of CuCo_2_O_4_. (D) Magnified SEM image of g-C_3_N_4_/CuCo_2_O_4_. (E) TEM image of CuCo_2_O_4_ nanopetals. (F) HRTEM image of one CuCo_2_O_4_ nanopetal.

Although CuCo_2_O_4_ materials are commonly discussed in the literature, the innovative and intriguing nanoflower g-C_3_N_4_/CuCo_2_O_4_ binder-free electrode on three–dimensional nickel foam stands out. Fig. 2A presents SEM views of the surface structure of nickel foam before and after acid treatment. It can be seen that there are distinct grain boundaries in both images, while the surface of the nickel foam after acid treatment is rougher than that without acid treatment. Notably, the rough surface created by acid treatment is essential for the nucleation process of metal ions. When the ion concentration surpasses the threshold necessary for nucleation, the resulting concentration supersaturation leads to the continuous precipitation of CuCo_2_O_4_ crystal nuclei on the rough surface of the nickel foam, serving as existing defect sites. Fig. 2B is a SEM image of CuCo_2_O_4_-grown nickel foam, showing that the surface of the nickel foam is coated densely with flower-like CuCo_2_O_4_ nanostructures. As shown in Fig. 2C, the distinctive flower-like CuCo_2_O_4_ with a diameter of approximately 5 μm consists of nanopetals. Fig. 2D is an SEM image of the flower-like structure of g-C_3_N_4_/CuCo_2_O_4_, showing that the few g-C_3_N_4_ layers are clustered together at the tips of the petals. This structure, obtained through secondary hydrothermal treatment, is stable and firmly adhered to the pristine nickel foam. The TEM image presented in Fig. 2E illustrates the morphology of the nanopetals scraped from a CuCo_2_O_4_ flower. As shown in Fig. 2F, the lattice fringes with interplanar distances of 0.195 and 0.454 nm correspond to the (400) and (111) crystal planes of CuCo_2_O_4_, respectively.

**Fig. 3:**
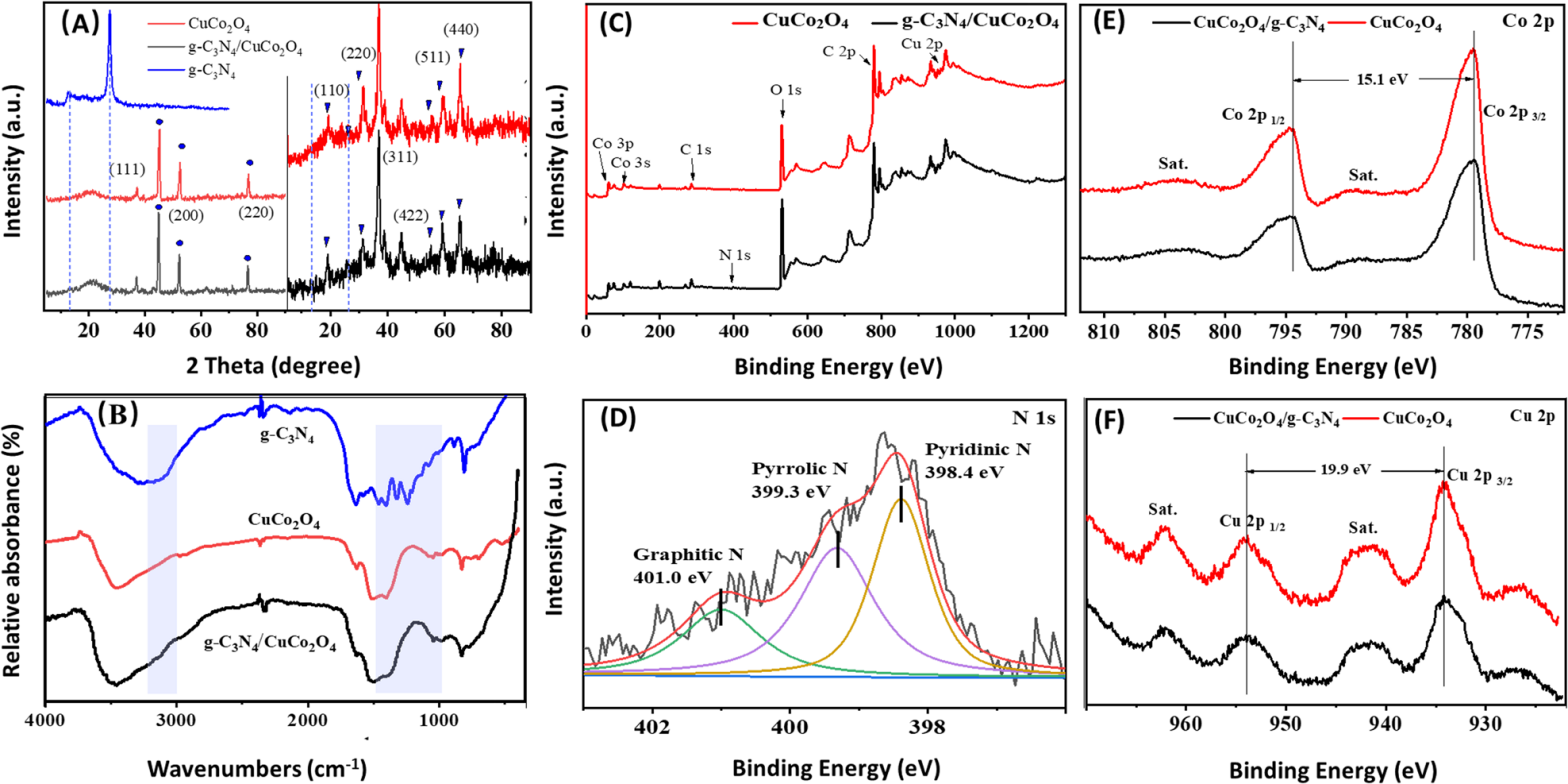
Spectral characterizations of the samples. (A) XRD patterns of CuCo_2_O_4_ and g-C_3_N_4_/CuCo_2_O_4_. (B) FTIR spectra of g-C_3_N_4_, CuCo_2_O_4_ and g-C_3_N_4_/CuCo_2_O_4_. (C) Survey XPS. (D) N 1s XPS of g-C_3_N_4_/CuCo_2_O_4_. (E) Co 2p and (F) Cu 2p XPS of CuCo_2_O_4_ and g-C_3_N_4_/CuCo_2_O_4_.

The phase structures of CuCo_2_O_4_ and g-C_3_N_4_/CuCo_2_O_4_ were analyzed using XRD. As shown in the left-hand panel of Fig. 3A, three sharp XRD peaks (indicated by “•”) are observed at 45.0°, 52.5°, and 76.8°, corresponding to the strong background of Ni (111), (200), and (220) planes originating from the nickel foam substrate. To observe the minor peaks more clearly, the *y*-axis is zoomed in to eliminate interference from background peaks, as shown in the right-hand panel of Fig. 3A. For the XRD patterns of both CuCo_2_O_4_ and g-C_3_N_4_/CuCo_2_O_4_, all the minor diffraction peaks can be definitively attributed to CuCo_2_O_4_ (JCPDS no. 37-0878). The identified diffraction peaks at 19.3°, 31.4°, 36.9°, 55.8°, 59.5°, and 65.3° (denoted by “▼”) can be categorized as the (111), (220), (311), (422), (333), and (440) planes, respectively. Pure g-C_3_N_4_ theoretically exhibits two prominent diffraction peaks at 13.2° and 27.4°, corresponding to the (100) and (002) peaks of the graphitic phase (JCPDS no. 87-1526).^22^ However, no distinct peak related to g-C_3_N_4_ is detectable in the XRD pattern of the g-C_3_N_4_/CuCo_2_O_4_ sample, possibly due to the lower content of g-C_3_N_4_.

To confirm the presence of g-C_3_N_4_, FTIR spectra of pure g-C_3_N_4_, CuCo_2_O_4_, and g-C_3_N_4_/CuCo_2_O_4_ are illustrated in Fig. 3B. All samples exhibit vibrations corresponding to surface-adsorbed water (1644 and 3100 cm^−1^) and CO_2_ molecules (2380 cm^−1^). In the case of pure g-C_3_N_4_, the prominent peaks at 1248, 1325, 1408, 1462, and 1570 cm^−1^ correspond to typical stretching modes of aromatic sp^3^ C–N bonds, while the peak observed at 809 cm^−1^ is indicative of the breathing mode associated with triazine units. For CuCo_2_O_4_, notable peaks located at around 3473 and 3226 cm^−1^ are linked to the stretching of –OH, and the peak at 1625 cm^−1^ reflects the bending modes of water molecules. Additionally, the peaks at approximately 1521, 1405, and 1046 cm^−1^ correspond to νOCO_2_, νCO_3_, and νC=O, respectively.^23,24^ The spectra distinctly exhibit the in-plane and out-of-plane bending vibrations of CO_3_^2−^ at 826 and 704 cm^−1^.^25^ The spectrum of g-C_3_N_4_/CuCo_2_O_4_ exhibits broader and more pronounced FTIR peaks at 3157 and 1363 cm^−1^ compared to pure CuCo_2_O_4_, suggesting the successful incorporation of g-C_3_N_4_.^26^ Although no evidence of g-C_3_N_4_ is found in the XRD results, the presence of g-C_3_N_4_ in the composite is confirmed by the FTIR spectra through identifying the characteristic vibrational peaks.

The survey XPS spectra (Fig. 3C) show the presence of elements of the CuCo_2_O_4_ and g-C_3_N_4_/CuCo_2_O_4_ samples. It is obvious that the peak of N 1s is present in the spectrum of the g-C_3_N_4_/CuCo_2_O_4_ sample. Fig. 3D displays the N 1s fine spectrum of g-C_3_N_4_/CuCo_2_O_4_, illustrating three notable peaks at binding energies of 398.7, 399.9, and 401.4 eV, corresponding to specific nitrogen functionalities, namely pyridinic nitrogen, pyrrolic nitrogen, and graphitic nitrogen, respectively.^27,28^Fig. 3E and F present a comparison of the high-resolution XPS spectra of Co 2p and Cu 2p, respectively. In the Co 2p spectra, two distinct peaks at binding energies of 779.5 and 794.6 eV are indexed to Co 2p_3/2_ and Co 2p_1/2_, respectively. The peak separation of 15.1 eV confirms the simultaneous presence of both Co^3+^ and Co^2+^ oxidation states.^29^ Cu 2p spectra display two prominent peaks at binding energies of 934.2 and 954.1 eV, corresponding to Cu 2p_3/2_ and Cu 2p_1/2_, respectively. The spin energy difference of approximately 19.9 eV corroborates the presence of the Cu^2+^ oxidation state in CuCo_2_O_4_. Two shakeup satellite peaks are observed at higher binding energy positions compared to the main peaks. The two satellite peaks are indicative of the Cu^2+^ oxidation state marked by “sat”.^30^ A notable decrease in peak intensity can be observed after the compounding process, suggesting that the coverage of g-C_3_N_4_ has a significant impact on the electrode's surface characteristics, leading to a diminished signal in the XPS analysis.

**Fig. 4:**
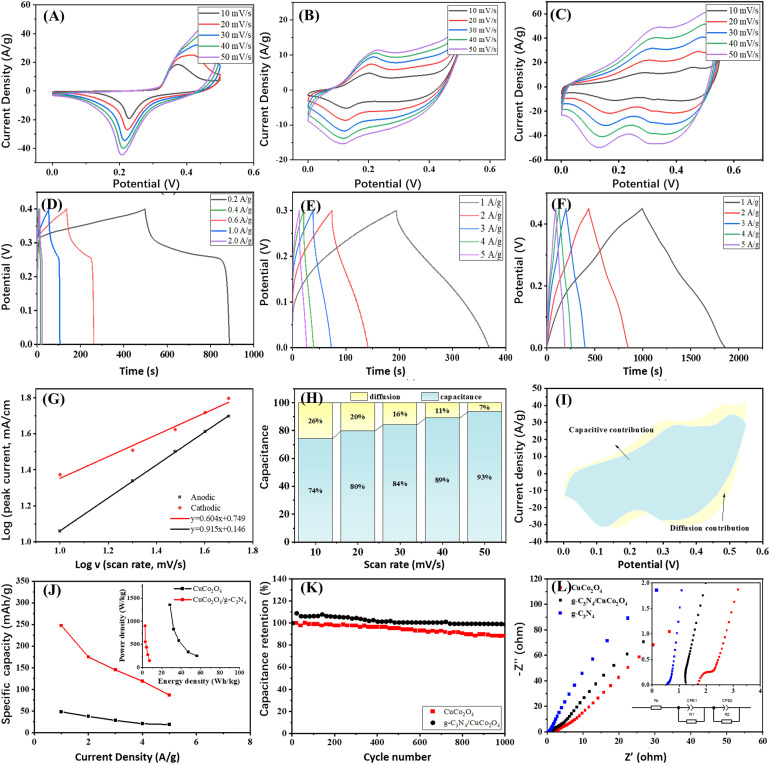
The electrochemical properties of g-C_3_N_4_, CuCo_2_O_4_ and g-C_3_N_4_/CuCo_2_O_4_. (A)–(C) The CV curves of g-C_3_N_4_ (A), CuCo_2_O_4_ (B), and g-C_3_N_4_/CuCo_2_O_4_ (C). (D)–(F) GCD curves of g-C_3_N_4_ (D), CuCo_2_O_4_ (E), and g-C_3_N_4_/CuCo_2_O_4_ (F). (G) The relationship between log(*v*) and log(*i*) of g-C_3_N_4_/CuCo_2_O_4_ for the redox peaks in the CV curves for a 6.0 M KOH electrolyte. (H) Histograms of the capacitance contributions from the CV curve of g-C_3_N_4_/CuCo_2_O_4_. (I) Relative contribution of capacitance. (J) The specific capacities of CuCo_2_O_4_ and g-C_3_N_4_/CuCo_2_O_4_ under different discharge current densities and inset showing energy density and power density. (K) The cycling stabilities of CuCo_2_O_4_ and g-C_3_N_4_/CuCo_2_O_4_ electrodes. (L) EIS spectra of g-C_3_N_4_, CuCo_2_O_4_ and g-C_3_N_4_/CuCo_2_O_4_ electrodes.

The electrochemical properties of the materials were assessed using CV and GCD studies. Fig. 4A–C present the CV curves of g-C_3_N_4_, CuCo_2_O_4_ and g-C_3_N_4_/CuCo_2_O_4_ at scan rates ranging from 10 to 50 mV s^−1^. The CV curves display distinct redox peaks, indicating reversible redox reactions involving the Cu^+^/Cu^2+^ and Co^3^+/Co^4+^ couples intricately linked with hydroxide ions (OH^−^) at the electrode–electrolyte interface. Fig. 4D–F depict the GCD curves obtained within a potential window at different current densities. The nonlinear characteristics of these curves suggest pseudocapacitive behavior. To further assess the reaction kinetics and charge storage processes in the g-C_3_N_4_/CuCo_2_O_4_ electrode, a power law approach is applied to represent the relationship between the peak current (*i*) and the scan rate (*v*) using formulas (4) and (5).

4 *i*_*v*_ = *k*_1_*v* + *k*_2_*v*^1/2^

5 *i*_*v*_/*v*^1/2^ = *k*_1_*v*^1/2^ + *k*_2_

The values of *k*_1_ and *k*_2_ can be determined by plotting *i*_*v*_/*v*^1/2^ against *v*^1/2^. As illustrated in Fig. 4G, the fitting results for *i versus v*^1/2^ reveal a strong linear correlation, suggesting that the charge storage mechanism is governed by the diffusion of electrolyte ions, thereby confirming the battery-type characteristics of g-C_3_N_4_/CuCo_2_O_4_.^31,32^ Ultimately, the fraction of current can be described for the charge storage process controlled by both diffusion and capacitance. The percentages of the capacitance-controlled contribution at varying sweep rates of 10, 20, 30, 40, and 50 mV s^−1^ are calculated to be 74%, 80%, 84%, 89%, and 93% of the total capacitance, respectively (Fig. 4H). Fig. 4I illustrates the CV profile of the g-C_3_N_4_/CuCo_2_O_4_ electrode at a sweep rate of 30 mV s^−1^, which is categorized into capacitance-controlled (blue region) and diffusion-controlled (yellow region) contributions. The proportion of the capacitance-controlled contribution relative to the overall performance is 84% at 30 mV s^−1^. The results indicate that at lower sweep rates, the diffusion-controlled process significantly influences the overall electrochemical behavior, implying that g-C_3_N_4_/CuCo_2_O_4_ facilitates the easy penetration of OH^−^ ions.^33^ In marked contrast, at higher sweep rates, the capacitance's contribution becomes more pronounced due to the constrained intercalation of electrolyte ions.^34^ The high rate capability of the g-C_3_N_4_/CuCo_2_O_4_ electrode may be attributed to the combined effects of both capacitance-controlled and diffusion-controlled contributions that enhance the redox reactions.

The pure g-C_3_N_4_ exhibits low specific capacity and rate property due to its limited electrical conductivity. Under current densities of 0.02, 0.04, 0.05, 1, 2, and 3 A g^−1^, the specific capacities are 20.6, 18.6, 16.7, 13.8, 11.3, and 9.75 mA h g^−1^, respectively. In contrast, CuCo_2_O_4_ and g-C_3_N_4_/CuCo_2_O_4_ demonstrate much higher performance. At different discharge current densities of 1, 2, 3, 4, and 5 A g^−1^, the CuCo_2_O_4_ electrode demonstrates specific capacitance values of 48.3, 37.6, 28.4, 20.7, and 18.7 mA h g^−1^, while the g-C_3_N_4_/CuCo_2_O_4_ electrode exhibits significantly higher specific capacitance values of 247.5, 175, 145, 119.2 and 87.0 mA h g^−1^, respectively (Fig. 4J). At any charging rate, the specific capacitance of g-C_3_N_4_/CuCo_2_O_4_ is at least triple that of pure CuCo_2_O_4_, suggesting a synergistic effect between g-C_3_N_4_ and CuCo_2_O_4_. The device achieves an energy density of 56.25 W h kg^−1^ and a peak power density of 1.38 kW kg^−1^, as shown in the inset of Fig. 4J. Fig. 4K shows the results of cycling stability tests. After 1000 cycles at a current density of 5 A g^−1^, the capacitance retention for the CuCo_2_O_4_ electrode is approximately 88%, indicating a loss of function. In contrast, the g-C_3_N_4_/CuCo_2_O_4_ electrode demonstrates a capacitance retention of 98%, representing an improvement of 10% over the CuCo_2_O_4_ electrode. This improvement is comparable with that of CuCo_2_O_4_-based nanocomposites reported in the literature.^16,35^ The improved stability can be attributed to the effective interaction between the uniformly distributed CuCo_2_O_4_ nanoflowers and few-layer g-C_3_N_4_, facilitating electron transport and mitigating the detrimental effects of voltage drop, particularly under high-rate operating conditions. This enhancement in stability is vital for optimizing performance and ensuring reliability in various applications.

To examine the charge transport dynamics of electrodes within the electrolyte, EIS analysis was performed on the CuCo_2_O_4_ and g-C_3_N_4_/CuCo_2_O_4_ electrodes, as shown in Fig. 4L. Incorporating g-C_3_N_4_ into CuCo_2_O_4_ results in a steeper slope in the EIS diagrams of the electrodes, indicating that the kinetics of the ion diffusion process is enhanced. The equivalent circuit model is fitted to evaluate the performance of the supercapacitor electrode (inset in Fig. 4L). The incorporation of g-C_3_N_4_ narrows the band gap, leading to enhanced conductivity of the electrode through decreasing the charge transfer resistance (*R*_ct_) and series resistance (*R*_s_).

A notable constraint in the commercial advancement of supercapacitors is the low energy density. As a result, considerable research initiatives have focused on improving the energy density of supercapacitors while preserving their high power density. Typically, supercapacitors are divided into symmetric and asymmetric types, depending on the electrode materials utilized for the negative and positive electrodes. Asymmetric devices have the ability to store a significantly greater amount of charge across a wider spectrum of operating potentials compared to their symmetric counterparts. This enhanced capacity is due to the utilization of distinct electrode materials, enabling the operating voltage of the device to surpass the thermodynamic decomposition voltage of water (1.2 V). Thus, we configure an active carbon (AC) electrode as the negative electrode and the g-C_3_N_4_/CuCo_2_O_4_ electrode as the positive electrode to construct an asymmetric supercapacitor.

**Fig. 5:**
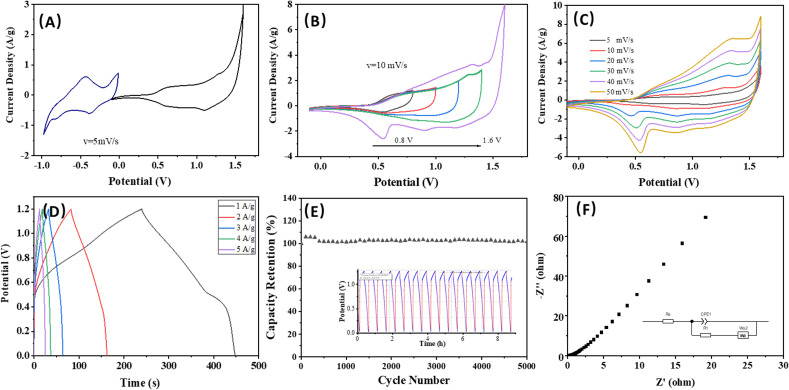
The electrochemical properties of asymmetric device (g-C_3_N_4_/CuCo_2_O_4_ with AC). (A) The CV curves of g-C_3_N_4_/CuCo_2_O_4_ and AC electrodes at a scan rate of 5 mV s^−1^. (B) The potential window variation for the upper potential windows for the asymmetric device. (C) The CV curves at different scan rates from 5 to 50 mV s^−1^. (D) The GCD curves of the asymmetric device. (E) The capacity retention after 5000 cycles. (F) Nyquist plot.

Fig. 5A illustrates the CV curves for the asymmetric device at a scan rate of 5 mV s^−1^ following charge balancing. In Fig. 5B, the CV profile is obtained across various voltage windows to identify the stable operating voltage of the asymmetric device. Notably, the working voltage of the asymmetric supercapacitor can be increased to 1.6 V when using an aqueous KOH electrolyte, surpassing that of conventional aqueous electrolyte AC supercapacitors. Fig. 5C depicts the representative CV curves of the device at different scan rates within a voltage range of −0.1 to 1.6 V. The shapes of all the CV curves are nearly identical, demonstrating excellent rate capability, high reversibility, and minimal internal resistance of the device. The GCD curves of the asymmetric device are shown in Fig. 5D, displaying that the device achieves a high energy density of 11.53 W h kg^−1^ and power density of 3.63 kW kg^−1^. Fig. 5E reveals that the device successfully retained 98% of its specific capacitance after undergoing 5000 cycles at a current density of 5 A g^−1^. This significant retention of capacitance not only demonstrates the device's strong performance but also confirms its excellent cycling stability, suggesting that it can reliably function over extended periods, making it a promising candidate for practical applications in energy storage solutions. The EIS measurement reaffirms the minimal internal resistance and the low charge/mass transfer resistance of the asymmetric device (Fig. 5F), which contributes to its good supercapacitance performance.

## 4. Conclusions

In conclusion, flower-like g-C_3_N_4_/CuCo_2_O_4_ structures have been successfully established by decorating g-C_3_N_4_ on CuCo_2_O_4_ nanostructures. The integration of g-C_3_N_4_ into CuCo_2_O_4_ electrodes has demonstrated significant improvements in electrochemical performance. Through CV and GCD analyses, the g-C_3_N_4_/CuCo_2_O_4_ heterostructure exhibits enhanced specific capacitance values and cycling stability compared to the unmodified CuCo_2_O_4_ electrode. Furthermore, EIS testing reveals that the incorporation of g-C_3_N_4_ leads to reduced charge transfer resistance and series resistance, indicating improved kinetics of ion diffusion and enhanced conductivity. These findings highlight the crucial role of the g-C_3_N_4_/CuCo_2_O_4_ structure in promoting extensive redox reactions, fast kinetics, and sufficient active sites for electrochemical reactions. These outstanding results indicate the significant potential applications as low-cost electrode materials. By employing a comparable approach for the precise synthesis of mesostructured metal oxides and g-C_3_N_4_, the efficiency of these advanced energy storage electrode materials can be enhanced, setting the stage for future advancements.

## Data availability

The data supporting the findings of this study are available within the article and further inquiries can be directed to the corresponding authors.

## Author contributions

Conceptualization, Y. F.; methodology, L. M., Y. F., S. Y.; validation, S. H., X. H.; formal analysis, L. M.; resources, L. M., S. H., X. H.; data curation, L. M., Y. F., S. Y.; writing – original draft, L. M., Y. F.; writing – review & editing, L. M., Y. F.; investigation, L. M., Y. F., S. Y., S. Z., S. H., X. H.; visualization, L. M.; supervision, L. M.; funding acquisition, L. M., Y. F.

## Conflicts of interest

The authors declare no conflict of interest.
